# Validation of Nanoparticle Response to the Sound Pressure Effect during the Drug-Delivery Process

**DOI:** 10.3390/polym12010186

**Published:** 2020-01-10

**Authors:** Mohamed Abbas, Mohammed Alqahtani, Ali Algahtani, Amir Kessentini, Hassen Loukil, Muneer Parayangat, Thafasal Ijyas, Abdul Wase Mohammed

**Affiliations:** 1 Electrical Engineering Department, College of Engineering, King Khalid University, Abha 61421, Saudi Arabia; hloukil@kku.edu.sa (H.L.); mparayangat@kku.edu.sa (M.P.); ithafasal@kku.edu.sa (T.I.); abdulwase@kku.edu.sa (A.W.M.); 2 Computers and Communications Department, College of Engineering, Delta University for Science and Technology, Gamasa 35712, Egypt; 3 Radiological Sciences Department, College of Applied Medical Sciences, King Khalid University, Abha 61421, Saudi Arabia; mosalqhtani@kku.edu.sa; 4 Mechanical Engineering Department, College of Engineering, King Khalid University, Abha 61421, Saudi Arabia; alialgahtani@kku.edu.sa (A.A.); akessentini@kku.edu.sa (A.K.); 5 Research Center for Advanced Materials Science (RCAMS), King Khalid University, P.O. Box 9004, Abha 61413, Saudi Arabia; 6 Laboratory of Electromechanical Systems (LASEM), National Engineering School of Sfax, University of Sfax, Route de Soukra km 4, Sfax 3038, Tunisia; 7 Nabeul’s Foundation Institute for Engineering Studies, University of Carthage, IPEIN, Nabeul 8000, Tunisia; 8 Electronics and Information Technology Laboratory, University of Sfax, National Engineering School of Sfax, Sfax 3038, Tunisia

**Keywords:** sound pressure interaction, nanoparticles, drug delivery, composite nanostructures, acceleration, particle displacement

## Abstract

Intravenous delivery is the fastest conventional method of delivering drugs to their targets in seconds, whereas intramuscular and subcutaneous injections provide a slower continuous delivery of drugs. In recent years, nanoparticle-based drug-delivery systems have gained considerable attention. During the progression of nanoparticles into the blood, the sound waves generated by the particles create acoustic pressure that affects the movement of nanoparticles. To overcome this issue, the impact of sound pressure levels on the development of nanoparticles was studied herein. In addition, a composite nanostructure was developed using different types of nanoscale substances to overcome the effect of sound pressure levels in the drug-delivery process. The results demonstrate the efficacy of the proposed nanostructure based on a group of different nanoparticles. This study suggests five materials, namely, polyimide, acrylic plastic, Aluminum 3003-H18, Magnesium AZ31B, and polysilicon for the design of the proposed structure. The best results were obtained in the case of the movement of these molecules at lower frequencies. The performance of acrylic plastic is better than other materials; the sound pressure levels reached minimum values at frequencies of 1, 10, 20, and 60 nHz. Furthermore, an experimental setup was designed to validate the proposed idea using advanced biomedical imaging technologies. The experimental results demonstrate the possibilities of detecting, tracking, and evaluating the movement behaviors of nanoparticles. The experimental results also demonstrate that the lowest sound pressure levels were observed at lower frequency levels, thus proving the validity of the proposed computational model assumptions. The outcome of this study will pave the way to understand the interaction behaviors of nanoparticles with the surrounding biological environments, including the sound pressure effect, which could lead to the useof such an effect in facilitating directional and tactic movements of the micro- and nano-motors.

## 1. Introduction

In recent years, nanoparticles have attracted considerable interest for various restorative applications. Under a rotating attractive field (AMF), certain magnetic nanoparticles (MNPs) can create heat through hysteresis that can be exploited to treat malignant growth. Furthermore, MNP heating has been presented as a strategy for remote neuromodulation [1]. The destabilizing particles are added to earlier nanoparticle during spilling of the scattering through the acoustic gap; the instigated collection prompts the arrangement of fundamentally slender microstructures [2]. The hydrodynamic vehicle of ring polymers through adjusted channels is expected on the ring topology [3]. Geometric scaling depends on the ratio of the maximum radii of air pockets in the two setups, and fluid element scaling follows the Rayleigh scaling [4].

An acoustic field was incorporated into a projection-based stereolithography (PSL) framework to design diverse microparticles into thick parallel bends or systems in the fluid tar. The impact of acoustic field settings and assembling process parameters on the design was described [5]. Moreover, particles can energize Kelvin waves on the vortex fiber through a reverberation instrument regardless of their distance [6]. The arrangement of the Navier-Stirs condition for an incompressible liquid based on the following two numerical strategies was proposed: a direct numerical simulation (DNS) in the light of a ghastly component strategy; and a pitifully non-straight plan (WNF) in the light of a limited component technique [7]. The expansion of winding balances diminishes the quality and stream lucidness of the vortex shedding process, resulting in a decline in the created sound weight during acoustic reverberation excitation [8]. The trial results of the assimilation coefficient were compared with the forecast of a model involving five physical parameters [9]. In another report, the impact of mass thickness on the aerogel sound transmission misfortune was clarified [10]. Another study presented the hypothesis of magnetoacoustics by foreseeing the presence of second-consonant weight waves from attractive nanoparticles because of vitality retention from consistently adjusted exchanging attractive fields [11]. The acoustic levitation and swarm accumulations of the metal nanoparticles can be effectively acknowledged at low vitality and clinically adequate acoustic recurrence by emptying their nanostructures. The acoustic levitation and swarm collections of the metal nanoparticles can be effectively acknowledged at low vitality and clinically worthy acoustic recurrence through the emptying mechanism of nanostructures [12]. Furthermore, acoustically powered micro- and nano-motors with various functioning mechanisms have been tested; they hold great potential for varied clinical needs, including drug delivery, minimally invasive surgical procedures, and biosensing [13,14,15,16]. For instance, the use of a levitation force provided by directed asymmetrical gradients of ultrasound pulses has resulted in self-acoustophoresis of metallic rod-type micromotors [17].

In recent years, Polymer nanocomposites have attracted considerable attention from scientists, and they have been extensively utilized for various biomedical methods, including drug-delivery [18]. Polymer nanocomposites have several advantages over the conventional drug-delivery methods. They have unique properties improving the process of drug-delivery, including chemical functionalization, multifunctional capabilities, and huge interphase zone [19]. These nanostructured molecules can freely travel through the blood and reach the diseased cells. Furthermore, they have unique physical properties that distinguish them from other medium-sized molecules when dealing with these cells. However, similarly to conventional drug molecules, nanoparticles face a problem that hinders their speed in the blood. The motion of particles is resisted due to the interaction between pressure and sound generated by the movement. Although this reaction is relatively small compared to the other traditional medium-sized molecules, it can reduce their movement in the blood. As a result, the drug-delivery process is slow.

The purpose of this research is to conceptualize the structure of these nanoparticles. Through this synthesis, the molecules can overcome the effect of sound pressure levels resulting from their movement as they flow into the blood to reach the target cells. Moreover, an experimental method is proposed, and the results are discussed.

## 2. Materials and Methods

The proposed structure of the nanoparticle drug and the related computational model are described in detail. The experimental validation of the proposed nanotechnology-based drug-delivery system is presented using various medical ultrasound (US) machines. The US images are evaluated on physically simulated models by injecting Aluminum 3003-H18 particles.

### 2.1. Sound Pressure Computational Model of Nanoparticle Drug

**Figure 1:**
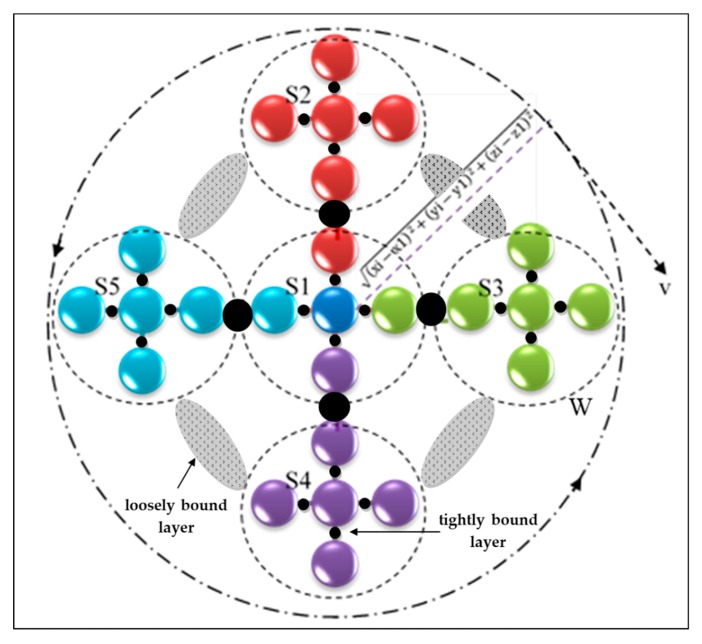
Nanocomposite structure of the proposed nanoparticles, (S1) molecular structure of the first material, (S2) molecular structure of the second material, (S3) molecular structure of the third material, (S4) molecular structure of the fourth material.

The proposed drug composition consists of a group of asymmetric nanoparticles that are intertwined in a circular form. The structure of pharmaceutical nanoparticles depends on the formation of molecules in multiple structures of similar composition, as shown in Figure 1. Each structure contains five different types of nanoparticles. The first pattern is centered in the middle of the structure and consists of five different nanoparticles. Figure 1 shows one of the five molecules in the middle, which is connected to the remaining four molecules regularly forming structure (S1). Each of these five elements is linked to another structure of the same type and labeled as S2, S3, S4, and S5. Here, Tsagaropoulos’ model interfaces were utilized in the proposed nanocomposite structure [20]. In this model, the joining of the nanoparticles in a polymer makes collaborations between the nanoparticles and the polymer chains, which are situated in the region of the nanoparticles. This cooperation causes locales with limited chain portability around the nanoparticles. Based on the provided model, each nanoparticle is encompassed by a tightly bound layer and a loosely bound layer. The loosely bound layer polymer displays its very own glass change, though the tightly bound layer doesnot take an interest in the glass progress. The structure is characterized by different attributes of these molecules, which can resist the motion of particles owing to the interaction between pressure and sound generated by their movement. This results in the rapid transfer of nanoparticles to the target. In order to clarify this concept, assume a nanoparticle pi (rpi) in three dimensions with radius (rp) at the center at point (*x*1, *x*2, *x*3) in each structure. Furthermore, x,y,z∈pi (rpi), where *i* is the position of the nanoparticle in the proposed architecture. Moreover, Green’s function is used to solve the differential equations of the proposed system. Since the proposed structure is acceptable, some of its parts separate from the other parts because of heterogeneity using the following equation:

(1) $$\begin{array}{ll} G\left(x,y\right)= & S\emptyset \left(\sqrt{{\left(xi-x1\right)}^{2}+{\left(yi-y1\right)}^{2}+{\left(zi-z1\right)}^{2}}\right)\\ & \;\;\;\;-\;(S\emptyset (\frac{\sqrt{\sum_{i=1}^{n}{\left(y_{i}-y1\right)}^{2}}}{\sqrt{{\left(xi-x1\right)}^{2}+{\left(yi-y1\right)}^{2}+{\left(zi-z1\right)}^{2}}}\overset{n}{\underset{i=1}{\sum }}(\left(xi-x1\right)\\ & \;\;\;\;-\;\frac{\left({\left(xi-x1\right)}^{2}+{\left(yi-y1\right)}^{2}+{\left(zi-z1\right)}^{2}\right)}{\sum_{i=1}^{n}{\left(y_{i}-y1\right)}^{2}}\left(yi-y1\right){)}^{2})) \end{array}$$

where S∅ is the singularity function and $\sqrt{{\left(xi-x1\right)}^{2}+{\left(yi-y1\right)}^{2}+{\left(zi-z1\right)}^{2}}$ is the radius of the nanoparticle at position *i*. Then, the solution to the proposed arbitrary linear differential is presented by the following equation:

(2) $$\begin{array}{ll} u\left(x\right)=-\overset{\infty }{\underset{B_{R}\left(0\right)}{\int }} & \frac{\partial }{\partial n_{y}}(S\emptyset \left(\sqrt{{\left(xi-x1\right)}^{2}+{\left(yi-y1\right)}^{2}+{\left(zi-z1\right)}^{2}}\right)\\ & -\;(S\emptyset (\frac{\sqrt{\sum_{i=1}^{n}{\left(y_{i}-y1\right)}^{2}}}{\sqrt{{\left(xi-x1\right)}^{2}+{\left(yi-y1\right)}^{2}+{\left(zi-z1\right)}^{2}}}\overset{n}{\underset{i=1}{\sum }}(\left(xi-x1\right)\\ & -\;\frac{\left({\left(xi-x1\right)}^{2}+{\left(yi-y1\right)}^{2}+{\left(zi-z1\right)}^{2}\right)}{\sum_{i=1}^{n}{\left(y_{i}-y1\right)}^{2}}\left(yi-y1\right){)}^{2}))){\mathrm{Sp}}_{y} \end{array}$$

where u(x) is the function of the arbitrary solution and Sp is the surface of the nanoparticle. In this context, *u* is given by Poisson’s formula that achieves the prescribed boundary values and ∃ is the closed contour of the nanoparticle. Let γ∈pi (rpi), z∈pi(rpi) and x∈pi(rpi). Then, the solution set in *x* and *z* dimensions is represented by Equations (3) and (4). For a small α>0, there is δ=δ(α) >0 such that |∃(y)−∃(γ)|<α.

(3) $$\begin{array}{ll} u\left(x\right)-\;\exists \left(\;\gamma \right)\;= & \overset{\infty }{\underset{\mathrm{pi}\left(\mathrm{rpi}\right)\left(\left|y-\gamma \right|<\delta \right)}{\int }}-\frac{\partial G\left(x,y\right)}{\partial y}|y\in \partial \mathrm{pi}\left(\mathrm{rpi}\right)(\exists \left(y\right)-\exists \left(\gamma \right)\;d{\mathrm{Sp}}_{y}\\ & +\overset{\infty }{\underset{\mathrm{pi}\left(\mathrm{rpi}\right)\left(\left|y-\gamma \right|\geq \delta \right)}{\int }}-\frac{\partial G\left(x,y\right)}{\partial y}|y\in \partial \mathrm{pi}\left(\mathrm{rpi}\right)\left(\exists \left(y\right)-\exists \left(\gamma \right)\right)\;d{\mathrm{Sp}}_{y} \end{array}$$

(4) $$\begin{array}{ll} u\left(z\right)-\;\exists \left(\;\gamma \right)\;= & \overset{\infty }{\underset{\mathrm{pi}\left(\mathrm{rpi}\right)\left(\left|z-\gamma \right|<\delta \right)}{\int }}-\frac{\partial G\left(z,y\right)}{\partial y}|y\in \partial \mathrm{pi}\left(\mathrm{rpi}\right)(\exists \left(y\right)-\exists \left(\gamma \right)\;d{\mathrm{Sp}}_{y}\\ & +\overset{\infty }{\underset{\mathrm{pi}\left(\mathrm{rpi}\right)\left(\left|z-\gamma \right|\geq \delta \right)}{\int }}-\frac{\partial G\left(z,y\right)}{\partial y}|y\in \partial \mathrm{pi}\left(\mathrm{rpi}\right)\left(\exists \left(y\right)-\exists \left(\gamma \right)\right)\;d{\mathrm{Sp}}_{y} \end{array}$$

It follows $\left|\int_{\mathrm{pi}\left(\mathrm{rpi}\right)\left(\left(\left|y-\gamma \right|+\left|z-\gamma \right|\right)<\delta \right)}^{\infty }H\left(x,y\right)\exists \left(y\right)-\exists \left(\gamma \right)dS{\;}_{y}\right|\leq \alpha$. Set M = max pi(rpi)∃(y). Then there is a $\delta^{\prime }>0$ such that

(5) $$H\left(x,y\right)<\frac{\alpha }{2M\omega_{n}{\left(\sqrt{{\left(xi-x1\right)}^{2}+{\left(yi-y1\right)}^{2}+{\left(zi-z1\right)}^{2}}\right)}^{n-1}}$$

If *x*, *z* and *y* satisfy $\left(\left|y-\gamma \right|+\left|z-\gamma \right|\right)<\delta^{\prime }$ and (|y−γ|+|z−γ|)>δ. Thus $\left|\int_{B_{R}\left(\left(\left|y-\gamma \right|+\left|z-\gamma \right|\right)<\delta \right)}^{\infty }H\left(x,y\right)\left(\exists \left(y\right)-\exists \left(\gamma \right)\right)\;dS{\;}_{y}\right|<\alpha$. By using Poisson’s formula, we have

(6) $$\begin{array}{ll} u\left(x\right)= & \frac{{\left(\sqrt{{\left(xi-x1\right)}^{2}+{\left(yi-y1\right)}^{2}+{\left(zi-z1\right)}^{2}}\right)}^{2}-{\left|x\right|}^{2}}{\omega_{n}\left(\sqrt{{\left(xi-x1\right)}^{2}+{\left(yi-y1\right)}^{2}+{\left(zi-z1\right)}^{2}}\right)}\overset{\infty }{\underset{\mathrm{pi}\left(\mathrm{rpi}\right)}{\int }}\exists \left(y\right)\\ & \;\;(1/(x^{2}+\;{\left(\sqrt{{\left(xi-x1\right)}^{2}+{\left(yi-y1\right)}^{2}+{\left(zi-z1\right)}^{2}}\right)}^{2}\\ & \;\;{-2x\left(\sqrt{{\left(xi-x1\right)}^{2}+{\left(yi-y1\right)}^{2}+{\left(zi-z1\right)}^{2}}\right)cos\;\delta )}^{n/2})\;dS{\;}_{y} \end{array}$$

To study the effect of the serum protein on the movement of the proposed structure, we use the equation that represents the relationship between the molecules and the serum protein [21]:

(7) $$N=\frac{4\pi {\left(\mathrm{Rm}+\mathrm{Ri}\right)}^{2}}{{\pi \mathrm{Ri}}^{2}}$$

Then,

(8) $$\mathrm{Rm}=-\mathrm{Ri}+\sqrt{\frac{N{\mathrm{Ri}}^{2}}{4\;}}$$

where *N* is the ideal number of binding sites for protein i, Rm is the molecular radius, and Ri is the protein radius that is represented as sphere. Figure 1 shows the architecture containing nine molecules along its diameter. Then, the radius of the structure is 18 Rm. Let us assume that all molecules have the same radius. Referring to the radius of the proposed structure, we have

(9) $$\sqrt{{\left(xi-x1\right)}^{2}+{\left(yi-y1\right)}^{2}+{\left(zi-z1\right)}^{2}}\;=\;8\;\mathrm{Rm}=8\left(-\mathrm{Ri}+\sqrt{\frac{N{\mathrm{Ri}}^{2}}{4\;}}\right)$$

Then, from Equation (6),

(10) $$\begin{array}{ll} u\left(x\right)= & \frac{{\left(8\left(-\mathrm{Ri}+\sqrt{\frac{N{\mathrm{Ri}}^{2}}{4\;}}\right)\right)}^{2}-{\left|x\right|}^{2}}{\omega_{n}\left(8\left(-\mathrm{Ri}+\sqrt{\frac{N{\mathrm{Ri}}^{2}}{4\;}}\right)\right)}\overset{\infty }{\underset{\mathrm{pi}\left(\mathrm{rpi}\right)}{\int }}\exists \left(y\right)\\ & \;\;\;(1/(x^{2}+\;{\left(8\left(-\mathrm{Ri}+\sqrt{\frac{N{\mathrm{Ri}}^{2}}{4\;}}\right)\right)}^{2}\\ & \;\;\;{-2x\left(8\left(-\mathrm{Ri}+\sqrt{\frac{N{\mathrm{Ri}}^{2}}{4\;}}\right)\right)cos\;\delta )}^{n/2})\;dS{\;}_{y} \end{array}$$

Equation (10) represents the effect of the serum protein on the velocity of the proposed structure in terms of the radii of protein particles.

In the two-dimensional case with $\omega_{n}\geq \partial \mathrm{pi}\;\left(\mathrm{rpi}\right)$, where ∃ is a potential function with respect to *x* or *y*, it is concluded that

(11) $$\begin{array}{ll} ∆u=\frac{1}{4\pi }∆\overset{\infty }{\underset{\exists }{\int }}f\left(y\right) & \frac{1}{\left|x-y\right|}\;dy\\ & +\overset{\infty }{\underset{\exists \emptyset \left(x,y\right)}{\int }}{∆}_{x}\exists \left(x,y\right)f\left(y\right)\;dy\\ & +\left(\overset{\infty }{\underset{\exists }{\int }}f\left(z\right)\frac{1}{\left|z-y\right|}\;dz+\overset{\infty }{\underset{\exists \emptyset \left(z,y\right)}{\int }}{∆}_{z}\exists \left(z,y\right)f\left(z\right)\;dz\right) \end{array}$$

Consider the following Euler equation to measure the effect of the pressure due to the flow of the composite nanoparticles in the blood:

(12) $$vt+\left(v.\nabla x\right)v+\frac{1}{\rho }\nabla x\;p=f\;$$

where *v* = (*v*1, *v*2, *v*3) is the speed vector. $v_{i}=v_{i}\left(x,t\right)$; *x* = (*x*1, *x*2, *x*3); *p* is the pressure, where *p* = (*x*, *t*); p is the density, where ρ=(x,t); and f=(f1,f2,f3) is the density of the external force. The total density of the composite nanoparticles is represented by the following equation:

(13) $$\rho =\;\frac{6\;ma}{\pi d^{3}}$$

where *ma* is the total mass of the composite nanoparticles and d is the diameter of the circular architecture of the composite nanoparticles. From Equations (12) and (13), the Euler equation to measure the effect of the pressure is represented in terms of the angular velocity of the composite nanoparticles:

(14) $$\begin{array}{l} \nabla x\;p\\ =\frac{6\;ma}{\pi {\left(2\sqrt{{\left(xi-x1\right)}^{2}+{\left(yi-y1\right)}^{2}+{\left(zi-z1\right)}^{2}}\right)}^{3}}\\ (f-w\;\sqrt{{\left(xi-x1\right)}^{2}+{\left(yi-y1\right)}^{2}+{\left(zi-z1\right)}^{2}}t\\ +\left(w\sqrt{{\left(xi-x1\right)}^{2}+{\left(yi-y1\right)}^{2}+{\left(zi-z1\right)}^{2}}.\nabla x\right)w\sqrt{{\left(xi-x1\right)}^{2}+{\left(yi-y1\right)}^{2}+{\left(zi-z1\right)}^{2}}) \end{array}$$

By considering the conservation mass, the following equation is considered in terms of angular velocity:

(15) $$\begin{array}{l} \frac{6\;ma}{\pi {\left(2\sqrt{{\left(xi-x1\right)}^{2}+{\left(yi-y1\right)}^{2}+{\left(zi-z1\right)}^{2}}\right)}^{3}}t\\ +\left(w\sqrt{{\left(xi-x1\right)}^{2}+{\left(yi-y1\right)}^{2}+{\left(zi-z1\right)}^{2}}.\nabla x\right)\frac{6\;ma}{\pi {\left(2\sqrt{{\left(xi-x1\right)}^{2}+{\left(yi-y1\right)}^{2}+{\left(zi-z1\right)}^{2}}\right)}^{3}}\\ +\frac{6\;ma}{\pi {\left(2\sqrt{{\left(xi-x1\right)}^{2}+{\left(yi-y1\right)}^{2}+{\left(zi-z1\right)}^{2}}\right)}^{3}}\nabla xw\sqrt{{\left(xi-x1\right)}^{2}+{\left(yi-y1\right)}^{2}+{\left(zi-z1\right)}^{2}}\\ =0 \end{array}$$

Assume that the blood is compressible and the following state function is applied p=p(ρ) where $p^{\prime }\left(\rho \right)>0$ if ρ>0. Then, the system is represented by the following applied equation:

(16) $$\begin{array}{l} w\sqrt{{\left(xi-x1\right)}^{2}+{\left(yi-y1\right)}^{2}+{\left(zi-z1\right)}^{2}}t\\ +\left(w\sqrt{{\left(xi-x1\right)}^{2}+{\left(yi-y1\right)}^{2}+{\left(zi-z1\right)}^{2}}.\nabla \right)v\\ +\frac{1}{\frac{6\;ma}{\pi {\left(2\sqrt{{\left(xi-x1\right)}^{2}+{\left(yi-y1\right)}^{2}+{\left(zi-z1\right)}^{2}}\right)}^{3}}}p^{\prime }\left(\frac{6\;ma}{\pi {\left(2\sqrt{{\left(xi-x1\right)}^{2}+{\left(yi-y1\right)}^{2}+{\left(zi-z1\right)}^{2}}\right)}^{3}}\right)\nabla p\\ =f \end{array}$$

### 2.2. Experimental Validation of the Proposed Nanotechnology-Based Drug-Delivery System

**Figure 2:**
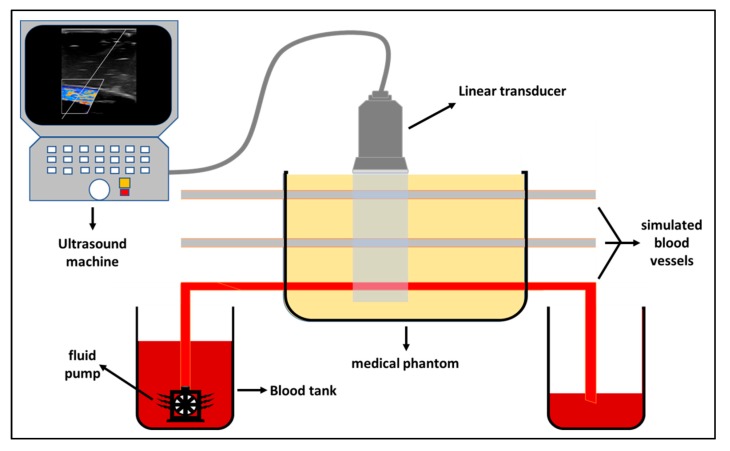
Schematic of the experimental setup, which shows the bespoke tissue-like phantom, connected blood tanks, and the type of the ultrasound transducer.

The experimental validation of the proposed nanotechnology-based drug-delivery system was performed using various medical ultrasound (US) machines. The US images produced were evaluated on physically simulated models by injecting Aluminum 3003-H18 particles (i.e., nanoscale fragments) intravenously. The experimental preparation included the development of a prototype US medical phantom with comparable characteristics of human soft tissues in terms of the interaction of US waves with tissue [22]. The US tissue-like phantom was developed using transparent silicone rubber and fully mixed with other scattering materials (Al_2_O_3_ and a cosmetic powder) to mimic the texture and color of human soft tissues. The ratios for mixing these prototype phantom components were 97.52% of silicone rubber, 0.26% of face cosmetic powder, and 2.22% of Al_2_O_3_ [23]. The mixture was allowed to slowly fill a Perspex container (length = 10 cm, width = 6 cm, and depth = 8 cm) to avoid the formation of air bubbles within the tissue-like phantom layers. Three silicon tubes (8 mm in diameter) were embedded in the prototype phantom at different depths (20 mm, 40 mm, and 60 mm) to simulate blood vessels at different conditions inside the human body. The mixture with the embedded tubes was stored for 72 h at room temperature to ensure that the whole mixture was fully cured. A blood sample (400 mL) was mixed with 50 mg of Al 3003-H18. Furthermore, a 12V CD waterproof fluid pump was used to provide continuous pumping of the blood mixture through the constructed phantom (Figure 2). The blood flow through the simulated tubes was scanned and evaluated using a medical US machine (Philips EPIQ 7 Ultrasound Machine) and a conventional linear traducer was used for the scanning procedure [24].

## 3. Results and Discussion

This section presents the validation results of the novel sound pressure computational model using acoustic-structure interaction COMSOL application. Then, the experimental validation results of the proposed nanotechnology-based drug-delivery system are presented. The results of the computational model and the experimental results are discussed.

### 3.1. Validation of the Novel Sound Pressure Computational Model

**Figure 3:**
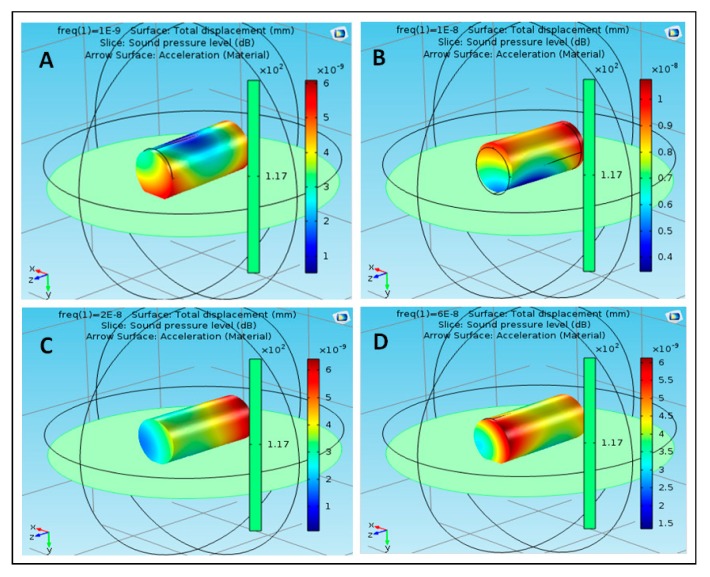
Effect of sound pressure interaction on polyimide nanoparticles in blood flow, (**A**) at 1 nHz, (**B**) at 10 nHz, (**C**) at 20 nHz, and (**D**) at 60 nHz.

**Figure 4:**
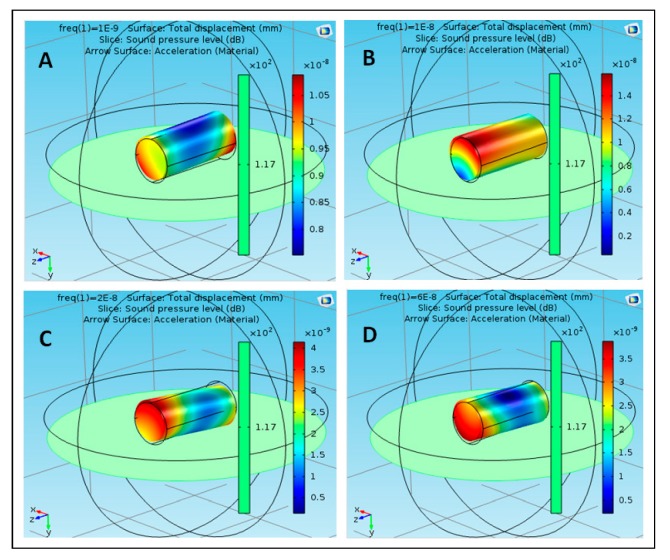
Effect of sound pressure interaction on Acrylic plastic nanoparticles in blood flow, (**A**) at 1 nHz, (**B**) at 10 nHz, (**C**) at 20 nHz and (**D**) at 60 nHz.

The proposed structure was simulated using acoustic–structure interaction COMSOL application [25]. Different materials were used to reflect the proposed structures (S1, S2 …… S5) for effective results. The materials that were used in the simulation included polyimide, acrylic plastic, Aluminum 3003-H18, Magnesium AZ31B, and polysilicon. These nanoparticles were selected based on their availability, cost, toxicity, and their physical properties and interaction with the targeted tissue inside the human body. S1 is composed of five particles, each of which is composed of a different material. The particle is in the center of the structure and is composed of polyimide. The remaining four molecules consist of four different materials, namely, acrylic plastic, Aluminum 3003-H18, Magnesium AZ31B, and polysilicon. S2 consists of five parts, all of which are similar and composed of acrylic plastic and directly linked to the structure through the part consisting of the acrylic plastic substance. Similarly, S3 is composed of Aluminum 3003-H18 and is directly connected to S1 through the nanoparticle of Aluminum 3003-H18. S4 is composed of Magnesium AZ31B and is connected to S1 through the nanoparticle of Magnesium AZ31B. S5 is composed of polysilicon and is connected to S1 through the nanoparticle of polysilicon. The effect of the sound pressure interaction on each nanoparticle of the material used in the simulation was studied with the nanoparticle analogy in the form of a cylindrical shape at frequencies of 1, 10, 20, and 60 nHz. Figure 3 shows the effect of sound pressure interaction on polyimide nanoparticles in the blood flow. The maximum value of sound pressure level (SPL) was 6 × 10^−9^ dB at 1 nHz, as shown in case (A), which did not have a strong effect on the movement of the nanoparticle due to the SPL effect on one end. The value of SPL of the nanoparticle of polyimide increased to 1 × 10^−8^ dB at 10 nHz, as shown in case (B), at both ends of the nanoparticle, which showed that SPL did not have a strong effect on its improvement. The value of SPL of the nanoparticle decreased to 6 × 10^−9^ dB at 20 nHz, which did not affect the motion of the nanoparticle, as shown in case (C). The value of SPL reached a maximum of 6 × 10^−9^ dB at 60 nHz as shown in case (D). It can be noted that at different frequencies of the nanoparticle of polyimide, the maximum value of SPL was noted at 10 nHz and the lowest value was noted at 20 nHz. Figure 4 shows the effect of sound pressure interaction on acrylic plastic nanoparticles in the blood flow. The extreme estimation of SPL was 1.1 × 10^−8^ dB at a repeat frequency of 1 nHz, as shown in case (A); in this case, the presence of the SPL impact was toward one side. The SPL of the nanoparticle of acrylic plastic increased to 1.6 × 10^−8^ dB at a repeat frequency of 10 nHz, as shown in case (B).

**Figure 5:**
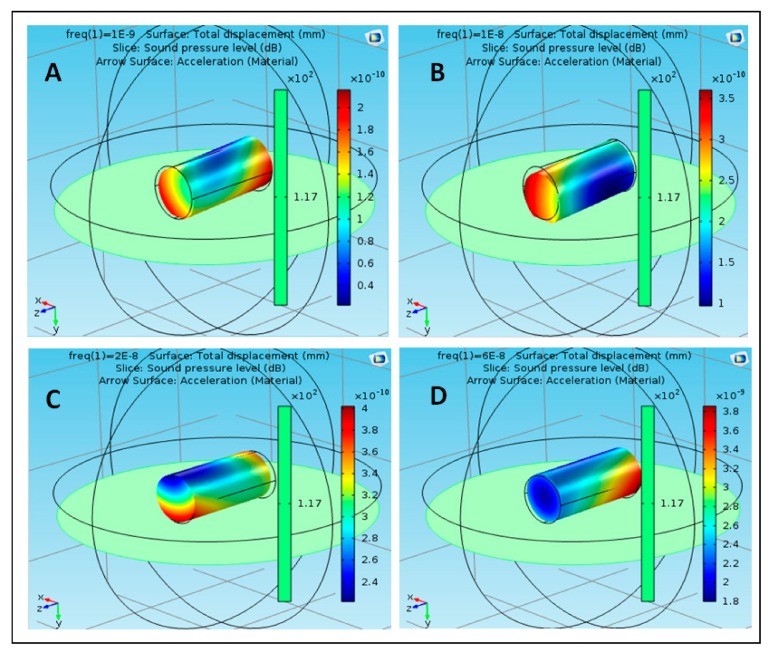
Effect of sound pressure interaction on Aluminum 3003-H18 nanoparticles in blood flow, (**A**) at 1 nHz, (**B**) at 10 nHz, (**C**) at 20 nHz and (**D**) at 60 nHz.

**Figure 6:**
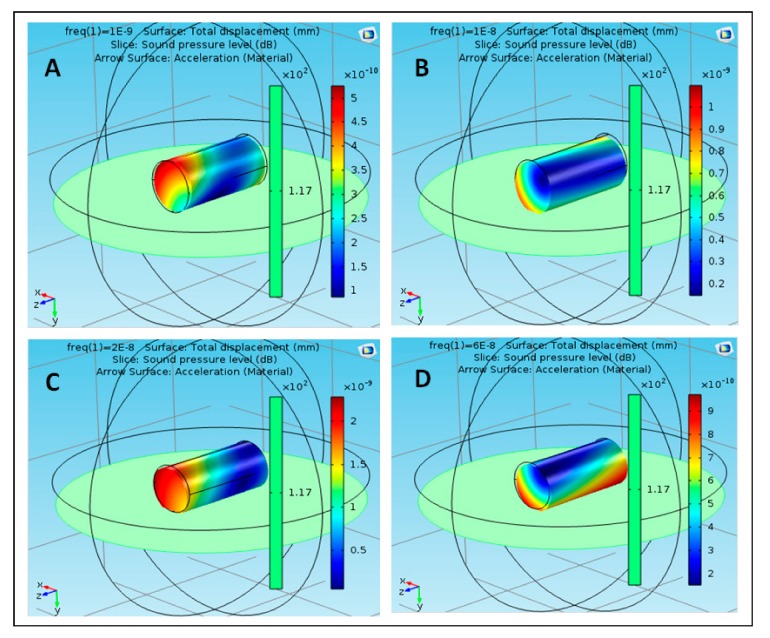
Effect of sound pressure interaction on Magnesium AZ31B nanoparticles in blood flow, (**A**) at 1 nHz, (**B**) at 10 nHz, (**C**) at 20 nHz and (**D**) at 60 nHz.

Owing to the nanoparticle development at 20 nHz, the value of SPL was 4 × 10^−9^ dB, which did not influence the movement of the nanoparticle. Case (D) demonstrates the estimation of SPL at a repeat frequency of 60 nHz, where it reached the extreme value of 4 × 10^−9^ dB. At various frequencies of the nanoparticle of acrylic plastic, the most extreme estimation of SPL was noted at 10 nHz. Figure 5 shows the effect of sound pressure interaction on Aluminum 3003-H18 nanoparticles in the blood flow. The maximum value of SPL was 2.2 × 10^−10^ dB at 1 nHz, as shown in case (A), which did not have a strong effect on the movement of the nanoparticle due to the SPL effect on both the ends. At a frequency of 10 nHz, the SPL of nanoparticle of Aluminum 3003-H18 increased to 3.6 × 10^−10^ dB, as shown in case (B). This effect was at only one end of the nanoparticle, which shows that SPL did not have a strong effect on its improvement. In case (D), the SPL of the nanoparticle decreased to 4 × 10^−10^ dB at 20 nHz, which did not affect the motion of the nanoparticle. Case (D) shows the value of SPL reached the maximum value of 3.8 × 10^−9^ dB at 60 nHz. At different frequencies of the nanoparticle of Aluminum 3003-H18, the maximum value of SPL was observed at 60 nHz. Figure 6 shows the effect of sound pressure interaction on Magnesium AZ31B nanoparticles in the blood flow. In the case of the nanoparticle of Magnesium AZ31B, the most extraordinary estimation of SPL was 5.2 × 10^−10^ dB at a repeat frequency of 1 nHz, as shown in case (A), which did not have a strong effect on the motion of the nanoparticle in view of the closeness of SPL sway along the nanoparticle. At a repeat frequency of 10 nHz, the value of SPL of nanoparticle of Magnesium AZ31B increased to 1.1 × 10^−9^ dB, as shown in case (B). This was along both the ends, which shows that SPL did not have a strong effect on its improvement. Owing to the nanoparticle improvement at 20 nHz, the SPL value reached 2.4 × 10^−9^ dB, which did not impact the development of the nanoparticle, as shown in case (C).

**Figure 7:**
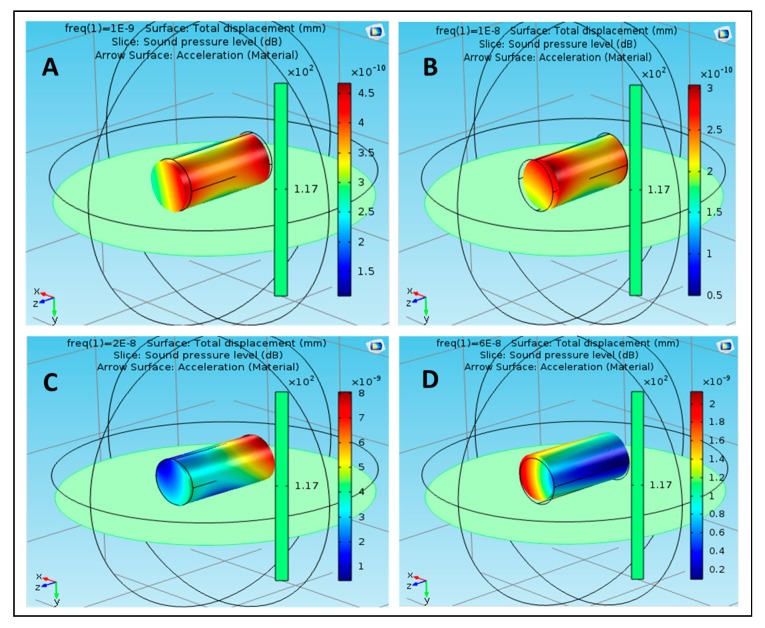
Effect of sound pressure interaction on Polysilicon nanoparticles in blood flow, (**A**) at 1 nHz, (**B**) at 10 nHz, (**C**) at 20 nHz and (**D**) at 60 nHz.

Case (D) shows the estimation of SPL at a repeat frequency of 60 nHz where SPL was the most outrageous at 10 × 10^−10^dB. At different frequencies of the nanoparticle of Magnesium AZ31B, the most outrageous estimation of SPL was noted at 20 nHz. Figure 7 shows the effect of sound pressure interaction on polysilicon nanoparticles in the blood flow. The maximum value of SPL was 4.6 × 10^−10^ dB at 1 nHz as shown in case (A). This value did not have a strong effect on the movement of the nanoparticle due to the appearance of the SPL effect on both the ends. Case (B) shows that at a frequency of 10 nHz, the value of SPL of the nanoparticle of polysilicon increased to 3 × 10^−10^dB. This was at only one end of the nanoparticle, which showed that SPL did not have a strong effect on its improvement. In case (C), SPL reached 8 × 10^−9^ dB at 20 nHz. Case (D) shows the value of SPL reached the maximum value of 2.2 × 10^−9^ dB at 60 nHz. That is, at different frequencies of the nanoparticle of polysilicon, the maximum value of SPL was observed at 20 nHz. In some cases, the value of SPL was noted at its lowest level around the body of the nanoparticles such as in the following cases: using polyimide nanoparticles in the blood flow at 1, 20, and 60 nHz.

**Figure 8:**
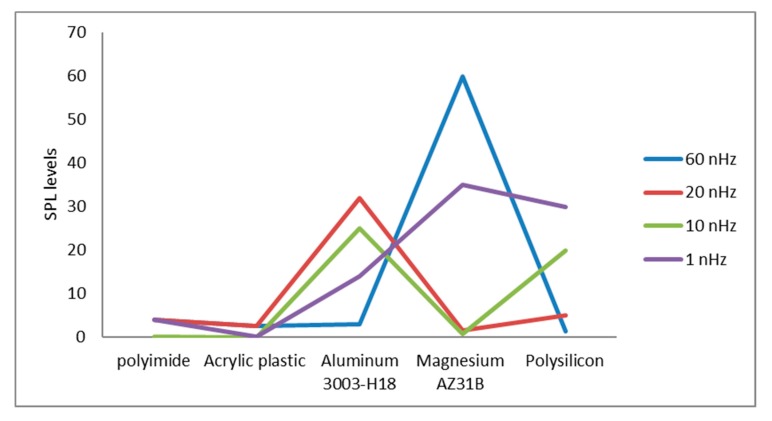
Average SP levels at different movement frequencies for the suggested materials.

Using acrylic plastic nanoparticles at 60 nHz; using Aluminum 3003-H18 nanoparticles at 1 nHz; using Magnesium AZ31B nanoparticles in the blood flow at 20 nHz; and using polysilicon nanoparticles in the blood flow at 10 nHz. The lowest level of sound pressure interaction on the nanoparticle terminals was achieved at lower levels of frequencies. Figure 8 shows the average SPLs at different movement frequencies for the suggested materials. The minimum average SPL was noted for the acrylic plastic material. All the minimum values of the sound pressure were obtained in all the ranges of the selected frequencies.

### 3.2. Experimental Validation of the Proposed Nanotechnology-Based Drug-Delivery System

Three phases of ultrasound scanning were conducted; in each phase, one of the simulated blood vessels was scanned and evaluated five times at different places through the length of the tube (i.e., at 1 cm, 3 cm, 5 cm, 7 cm, and 9 cm from the phantom’s edge). Each scan was composed of two scanning orientations, at all selected scanning areas, which show the simulated vessels along the longitudinal and transverse planes. This medical imaging experiment produced 30 sonographic images, and the peak systolic velocity (PSV) was recorded as an indication for the angular speed inside the simulated vessels, as the blood circulation in this experimental setup was isolated from the effect of irregular blood flow rate (i.e., pulses) through the use of a mono-type fluid pump. The PSV was recorded 10 times for each scanned simulated vessel and the recorded values were averaged. Furthermore, the presented sonographic images were qualitatively evaluated.

**Figure 9:**
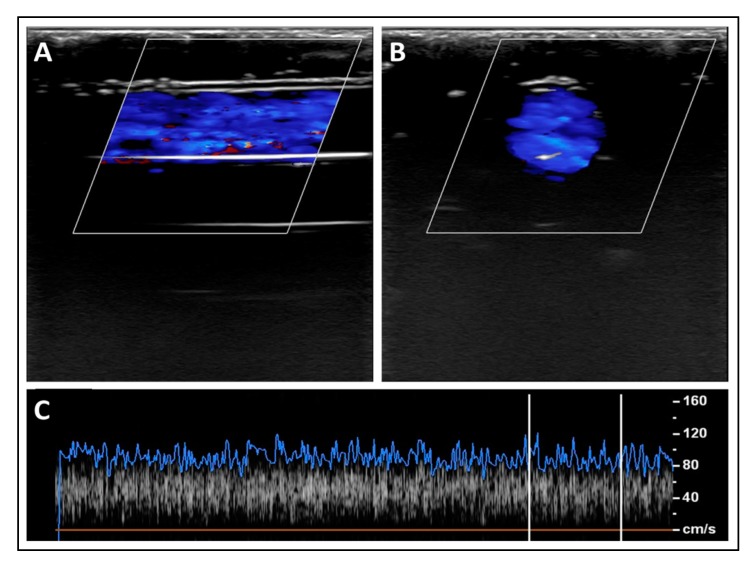
A sample of longitudinal (**A**) and transverse (**B**) scans for the superficial simulated blood vessel (depth = 20 mm). (**C**) A spectrum of the fitted blood speed’s readings over one scanning cycle.

**Figure 10:**
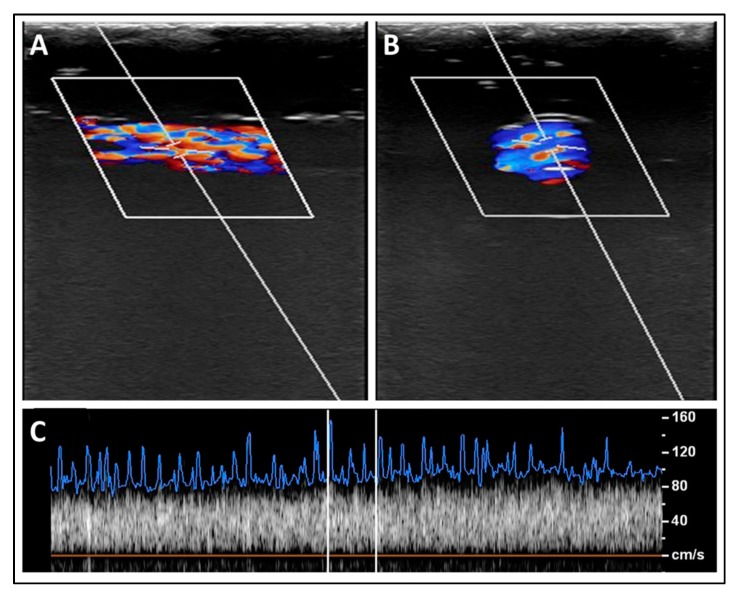
A sample of longitudinal (**A**) and transverse (**B**) scans for the medium-depth simulated blood vessel (depth = 40 mm). (**C**) A spectrum of the fitted blood speed’s readings over one scanning cycle.

**Figure 11:**
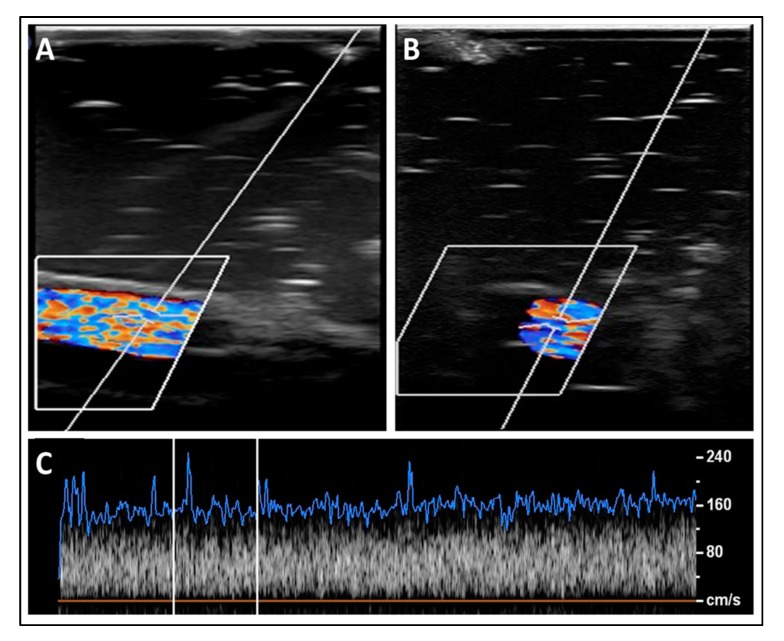
A sample of longitudinal (**A**) and transverse (**B**) scans for the deep simulated blood vessel (depth = 60 mm). (**C**) A spectrum of the fitted blood speed’s readings over one scanning cycle.

As seen in Figure 9, the blood mixture was pumped through the superficial simulated blood vessel and the average recorded speed was 124 ± 5.81 cm/s (Figure 9C). The aliasing effect was minimal, as a mono-color was visualized, which represented the one-direction flow of the blood inside the simulated vessels. The aliasing results from a direct effect of sampling added noise. In this case, aliasing relates to the sound of the particle movement. Hence, the level of aliasing is related to the sound pressure levels of the nanoparticles. However, the aliasing effect (i.e., multi-colored flow) became more pronounced when deeper simulated blood vessels were scanned, owing to the pressure provided by surrounding simulated tissues, which increased the stress on the outer shell of the Al nanoparticles and resulted in the angular spinning of these nanoparticles. This effect can be visualized as multi-colored flow inside the simulated blood vessels (Figure 10A,B). The stenosis in the deep simulated blood vessel provided by the outer pressure from the simulated tissues made the aliasing effect well-recognized (Figure 11A,B). The average recorded speeds in the medium-depth (40 mm) and the deep (60 mm) simulated blood vessels were 159 ± 7.36 cm/s and 212 ± 9.82 cm/s, respectively (Figure 10C and Figure 11C). The percentage differences between the superficial simulated blood vessel and the deeper blood vessels were 24.74% for the medium-depth simulated blood vessel and 52.38% for the deep imitated vessel, respectively.

The deep main vessels inside the human body are wider (such as the abdominal aorta and the inferior vena cava), and the vessels become narrower close to the superficial layer of the skin. Therefore, the aliasing effect could be a potential marker that can be used to trace nanoparticles for drug-delivery purposes, as the experimental results are in agreement with the computational models’ outcome.

Ultrasound technologies are challenged, as they have minimal clinical impact in quantifying the blood flow in small blood vessels; however, the recent advances in ultrasound technologies have paved the way to detect the blood microcirculation inside the human body. Furthermore, they could facilitate the possibility of tracing nanoparticles inside convoluted blood vessel networks [26]. Moreover, ultrasonic techniques for detecting and tracing nanoparticles could be enhanced with the rapid growth of using microbubble contrast agents, and their second generation that can be filled with heavy molecular weight particles could be combined with various nanoparticles, which enhances the capabilities of targeting specific tissues inside the human body [26]. The binding process of the microbubble contrast agent with biomarkers enriches the tumor angiogenesis evaluation and the future direction of these state-of-the-art techniques could improve the possibility and sensitivity of using different nanoparticles in detecting and staging tumors and in visualizing their blood microcirculation [27,28].

## 4. Conclusions

The use of nanotechnology in medicine is increasing rapidly. Nanoparticles encounter a number of sequential obstacles as they travel through the bloodstream to reach the affected cell, thus gradually reducing their movement. As a result, the drug transmission process is slow. One of the obstacles is the acoustic pressure levels created by the sound waves associated with the molecules as they circulate in the blood. This study aimed to achieve nanoparticle synthesis of drug molecules that act as catalysts for drug nanoparticles in overcoming acoustic pressure levels. The results demonstrate the potential of the proposed design. A weak effect of pressure levels was observed on both ends and around the body of nanoparticles. The research suggests the use of five material molecules, namely polyimide, acrylic plastic, Aluminum 3003-H18, Magnesium AZ31B, and polysilicon, owing to their lower cost and availability. The experimental validation method was proposed and conducted. The experimental results proved the possibility of evaluating the effect of sound pressure on the movement of nanoparticles. In addition, the results are in good agreement with those of the proposed computational model in that the lowest levels of sound pressure were noted at lower frequencies of the nanoparticles. Moreover, potential technologies for detecting and tracing nanoparticles for drug-delivery purposes were discussed. It is worth mentioning that employing advanced medical imaging technologies in nanoparticle detection and evaluation can positively contribute to various computational nanoparticle models in terms of proofing the concepts and validating the proposed models. Furthermore, the produced results could facilitate further evaluation of the sound pressure effect in terms of advancing the methods used for directional and tactic motions of micro- and nano-motors.
